# Individual capacity-building approaches in a global pharmaceutical systems strengthening program: a selected review

**DOI:** 10.1186/s40545-017-0104-z

**Published:** 2017-05-08

**Authors:** Niranjan Konduri, Megan Rauscher, Shiou-Chu Judy Wang, Tanya Malpica-Llanos

**Affiliations:** 1 Systems for Improved Access to Pharmaceuticals and Services (SIAPS) Program, Management Sciences for Health, Arlington, VA USA; 2 Pharmaceuticals and Health Technologies Group, Management Sciences for Health, Arlington, VA USA

**Keywords:** Human resources, Pre-service training, In-service training, Pharmaceutical systems, Pharmaceutical services, Capacity building

## Abstract

**Background:**

Medicines use related challenges such as inadequate adherence, high levels of antimicrobial resistance and preventable adverse drug reactions have underscored the need to incorporate pharmaceutical services to help achieve desired treatment outcomes, and protect patients from inappropriate use of medicines. This situation is further constrained by insufficient numbers of pharmaceutical personnel and inappropriate skill mix. Studies have addressed individual capacity building approaches of logistics, supply chain or disease specific interventions but few have documented those involving such pharmacy assistants/professionals, or health workers/professionals charged with improving access and provision of pharmaceutical services. We examined how different training modalities have been employed and adapted to meet country-specific context and needs by a global pharmaceutical systems strengthening program in collaboration with a country’s Ministry of Health and local stakeholders.

**Methods:**

Structured, content analysis of training approaches from twelve selected countries and a survey among conveniently selected trainees in Bangladesh and Ethiopia.

**Results:**

Case-based learning, practice and feedback, and repetitive interventions such as post-training action plan, supportive supervision and mentoring approaches are effective, evidence-based training techniques. In Ethiopia and Bangladesh, over 94% of respondents indicated that they have improved or developed skills or competencies as a result of the program’s training activities. Supportive supervision structures and mentorship have been institutionalized with appropriate management structures. National authorities have been sensitized to secure funding from domestic resources or from the global fund grants for post-training follow-up initiatives. The Pharmaceutical Leadership Development Program is an effective, case-based training modality that motivates staff to develop quality-improvement interventions and solve specific challenges. Peer-to-peer learning mechanisms than traditional didactic methods was a preferred intervention among high level government officials both within country and between countries.

**Conclusion:**

Interventions must involve local institutions in the design and delivery of content for both pre-service and in-service training as well as web-based methods where feasible. Such efforts would meet the changing demand in the pharmaceutical system, and promote the ownership of the human capacity development interventions. The cost-effective partnership with universities demonstrate that competency based pre-service training will prepare the future pharmaceutical workforce with a critical foundation of knowledge and skills required to meet the growing demand for patient-centered pharmaceutical services in resource-constrained countries.

**Electronic supplementary material:**

The online version of this article (doi:10.1186/s40545-017-0104-z) contains supplementary material, which is available to authorized users.

## Background

The Sustainable Development Goal 3.8 of the 2030 Agenda for Sustainable Development specifies that in order to achieve universal health coverage, national development strategies must include “access to safe, effective, quality and affordable medicines and vaccines for all” [1]. The Lancet commission on essential medicines for universal health coverage acknowledged that professionals such as pharmacists and prescribers as well as dispensers need specialized training and information to assure the appropriate use of medicines in the interest of patients and caregivers [2]. Beyond access, medicines use related challenges such as inadequate adherence [3], high levels of antimicrobial resistance [4] and the impact of preventable adverse drug reactions [5] have underscored the need to incorporate patient-oriented pharmaceutical services that help to achieve desired treatment outcomes and protect patients from harm [6, 7]. This situation is further constrained by insufficient numbers of pharmaceutical personnel and incomplete or inappropriate skill mix to respond to the needs of local populations [8, 9].

The Systems for Improved Access to Pharmaceuticals and Services program (the ‘program’) funded by the U.S. Agency for International Development (USAID) works in partnership with local governments and partners to build resilient pharmaceutical systems that deliver safe, timely, and quality pharmaceuticals and healthcare services, through a pharmaceutical systems strengthening approach [10, 11]. The program’s overall capacity building approach was adapted from Potter and Brough’s “Pyramid of Effective Needs” and includes nine interrelated components [12, 13]. The components are categorized into individual (performance capacity and personal capacity) and institutional (workload, facility, supervisory, support service, structural, systems, and role capacity). Potter and Brough assert that developing a common definition of capacity building is challenging because of different sociocultural settings. Instead, establishing a hierarchy of capacity-building needs is more useful for designing and implementing activities to address varied capacity gaps.

Pharmaceutical systems in resource-constrained countries are challenged by limited number of institutions for pharmaceutical training and lack of up-to-date training curricula. Using locally relevant and context specific training approaches, the program aims to build and augment individual capacity building interventions to ensure that the pharmaceutical health workforce has the right skill mix and distribution to meet population needs and the appropriate tools to do so. Published studies have addressed individual capacity building approaches of logistics, supply chain [14] or disease specific interventions [15]. However, to our knowledge, from a global program implementer perspective, few have documented the programmatic approach of building individual capacity involving pharmacy professionals, pharmacy assistants or health workers charged with improving access and provision of pharmaceutical services. The objective of this paper is to summarize the various approaches that have been implemented by the program in collaboration with a country’s Ministry of Health and local stakeholders, and examine the influence of the training on individual capacity, as defined by Potter and Brough. Using selected, specific country examples, we examined how different training modalities have been employed and adapted to meet country-specific context and needs.

## Methods

Twelve program implementing countries were selected for review based on internal funding availability during the study period June to December 2015: Angola, Bangladesh, Burundi, Cameroon, Democratic Republic of the Congo [DR Congo], Ethiopia, Mali, Namibia, Philippines, South Africa, Swaziland, and Ukraine. We retrieved documentation from the first 3 years of program implementation that was available between 2012 and 2015. We performed a structured, content analysis of training activities from the program’s topic specific technical reports, quarterly and annual reports. Internal reports from a one-off training event or multi-event training series were reviewed, which typically provide the training objectives and training methods employed. Country-specific technical reports that contained information on an intervention with a training component were reviewed. After desk review of project documents, each country or portfolio was contacted for the purpose of clarifying content or seeking elaboration on any training methodologies if not documented in sufficient detail. Through our country program, we conducted a survey among conveniently selected trainees in Bangladesh and Ethiopia due to the large number of pharmaceutical personnel trained (Additional file 1 provides details on the methodology). In-country program staff also conducted key informant interviews with 11 authorities in Bangladesh and 19 authorities in Ethiopia who were part of the training initiatives. Institutional permission was obtained from the Ministry of Health personnel based on our country program’s ongoing monitoring and evaluation mechanism.

## Results

The program’s individual capacity building approaches can broadly be grouped into three categories: pre-service, in-service and proven approaches for individual capacity building. Pre-service training is defined as activities that take place before a person starts a job that requires specific training [16], i.e., before a person “enters service” whereas in-service training is considered training of persons who are already employed, e.g., health care providers working in the public or private sector [17]. Drawing upon lessons learned from our predecessor Strengthening Pharmaceutical Systems program (2007–2011) [18], this paper presents the following proven approaches to individual capacity building as applied to either pre-service or in-service training approaches: supportive supervision [19, 20], team based and peer-to-peer learning mechanisms [21, 22], e-learning and blended learning [23]. The program has utilized blended learning models (a combination of face-to-face and virtually facilitated sessions) to scale up the delivery of training activities in a cost-effective, sustainable manner, especially in collaboration with local institutions.

### Pre-service training approaches

**Fig. 1:**
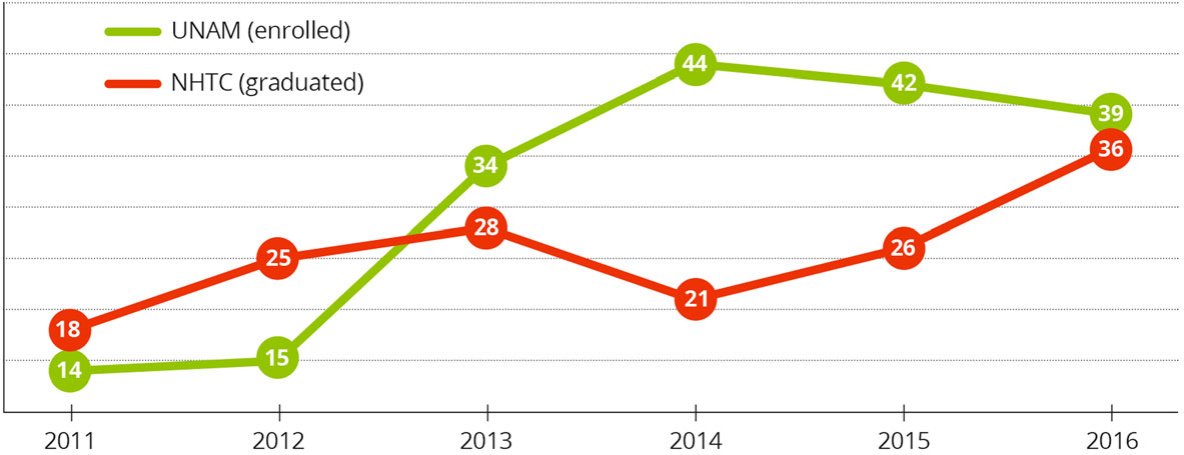
Number of pharmacist assistants graduating from the National Health Training Center, by year of graduation

A key impediment to the scale-up of pharmaceutical services to address the HIV burden in Namibia was the shortage of skilled pharmaceutical personnel, including pharmacists and pharmacy assistants [24]. To increase the number of qualified pharmaceutical professionals, the program supported two major pre-service activities: the development of a local Bachelor of Pharmacy (B Pharm) degree [25], and revamping accompanying training curricula and supporting the training efforts of the National Health Training Centre (Fig. 1) [26]. By 2014, the number of enrolled B Pharm students increased to 107, and the first 14 graduated received their B Pharm degree in 2015. Details on the process of establishing Namibia’s first school of pharmacy is available in the program website [27, 28]. A wide range of support to improve the quality of pre-service training for B Pharm students and pharmacist assistants was provided, including curriculum revision, recruitment of pharmacy lecturers, information technology assistance, accreditation and quality management system support [29, 30].

**Fig. 2:**
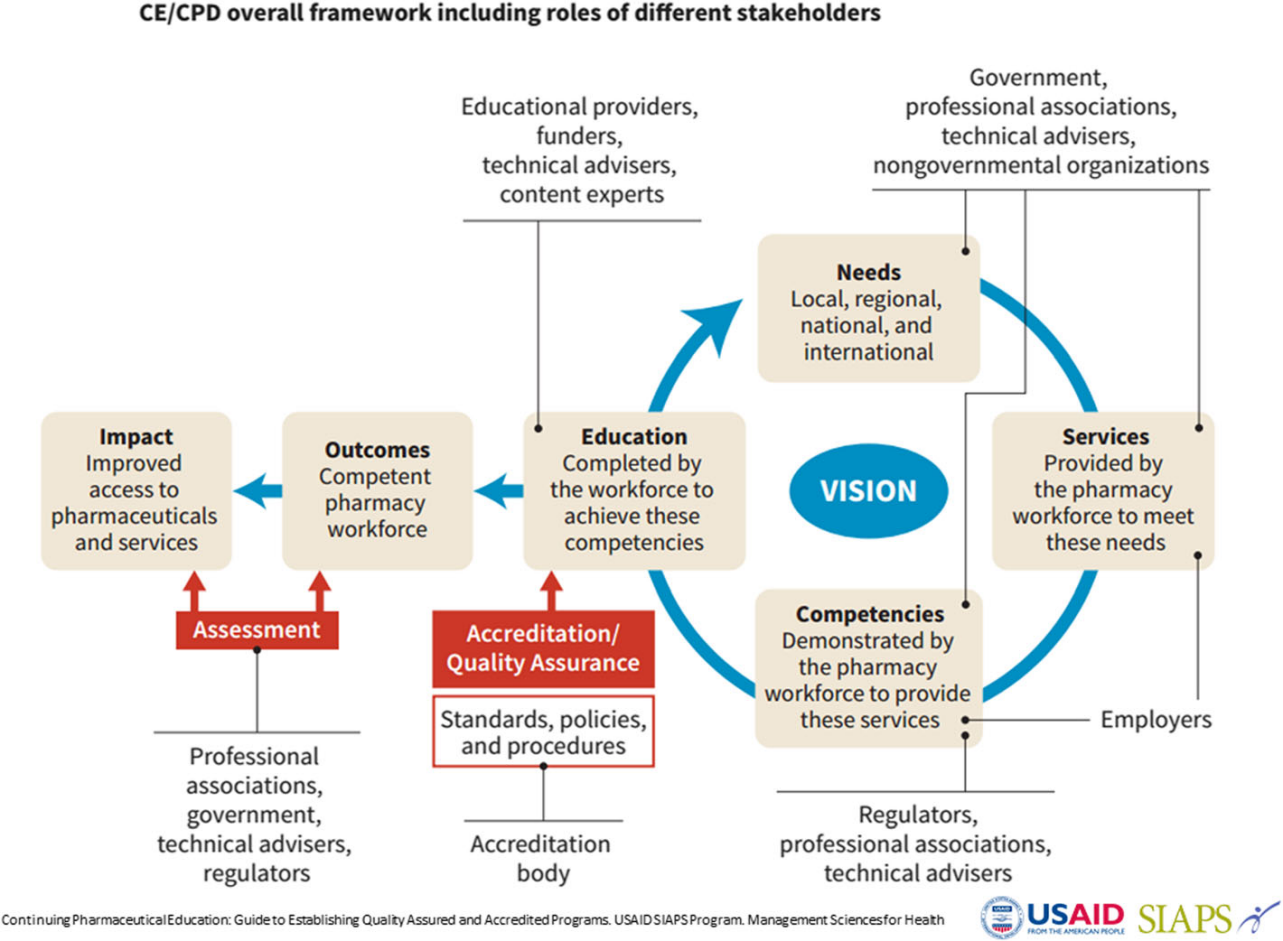
Continuing Education and Continuing Professional Development Framework, including Roles of Different Stakeholders

In DR Congo, the program’s partner, the Accreditation Council for Pharmaceutical Education (ACPE) [31], reviewed the pharmacy school curriculum at the University of Kinshasa and developed a road map for updating and implementing a revised curriculum that meets international standards [32]. DR Congo’s vision is to prepare competent pharmacists who can better address public health priorities, particularly in the areas of supply chain management, appropriate use of medicines, pharmaceutical services, pharmacovigilance and antimicrobial resistance. National stakeholders recognized this intervention as a high-impact activity with the results spinning off on the other three faculties of pharmacy in the country and the National Pharmacy Council, thereby influencing pharmacy education and practice in the whole country, including the private sector. Thereafter, the program supported the University of Kinshasa to develop a 5-year strategic plan to improve the coordination, monitoring, and evaluation of faculty operations and to develop a competency framework [33], which defines required cognitive, procedural, and behavioral competencies that graduated pharmacists should have upon completion of their degree (Fig. 2) [34].

This competency framework was a major step in overhauling current training curricula and recognized by the USAID’s Mission Director in DR Congo (quote): “*To improve the pharmaceutical sector, we must address the root of the problem, which is pharmacist training*” [35]. Table 1 summarizes key results in Namibia and DR Congo.

**Table 1:** Relevant Results (Namibia and DR Congo)

	As of December 2015	Key Results
Namibia	41% increase in the number of certified pharmacy personnel	• B Pharm and PA competency framework developed • Accreditation for B Pharm program and PA courses received • B Pharm program launched in 2011 • The majority (75%) of the pharmacist assistants reported overall satisfaction with their PA training at the NHTC • Over 90% of the employers and supervisors were satisfied with the pharmacist assistants’ performance at work
	97% of public health facilities are staffed with certified pharmacy personnel	
DR Congo	1103 persons trained in pharmaceutical management	• Pre-service competency framework developed with 5-year strategic plan
	2 health or allied health professional associations or councils receiving TA in pharmaceutical management education	

### In-service training approaches

Globally, as of December 2015, 35 in-service training curricula had been developed or revised in 11 countries with the program’s support. Nearly 39,000 individuals (31% female, 62% male) in over 24 countries (Table 2) had been trained in various aspects of pharmaceutical management, including: financing, leadership, regulatory systems, quality assurance, pharmaceutical care, medicine safety, antimicrobial resistance, and supply chain management.

**Table 2:** Number of Personnel Trained in Pharmaceutical Management, as of December 2015

Country/portfolio	Number trained
Angola	251
Bangladesh	15,594
Burundi	2890
Cameroon	741
Dominican Republic	2232
DR Congo	1103
Ethiopia	5110
Guinea	796
Latin America and Caribbean Amazon Malaria Initiative	554
Lesotho	438
Mali	1593
Mozambique	579
Namibia	501
Neglected tropical diseases core portfolio	35
Philippines	358
South Africa	1087
South Sudan	864
Swaziland	1092
Tuberculosis core portfolio	2695
Turkmenistan	22
Ukraine	280
West Africa Regional	120
Total	38,935

The program seeks to ensure that its capacity-building efforts address immediate country needs to empower and enable country governments and local institutions to develop, implement, and own the technical assistance and capacity-building efforts. Globally, as of December 2015, the program facilitated 394 local institutions or organizations to provide training or technical assistance in pharmaceutical management.

### Cascade training approaches

To strengthen sustainability efforts, the program often employs a cascade training approach to rapidly develop new knowledge or skills in specific pharmaceutical management topics and capacitate national staff in local institutions. In an effort to address the high burden of multidrug-resistant tuberculosis, the program and National Tuberculosis Program (NTP) staff piloted training on the e-TB Manager (web-based tool for managing all the information needed by national TB control programs) in three oblasts (states) of Ukraine, which resulted in a decision to scale up the use of e-TB Manager nationwide [36]. Given the country’s large size and the resource intensive nature of scale up, a training of trainers (TOT) approach was selected to more efficiently cascade the training across the country. The program engaged a local Ukrainian group specializing in adult learning techniques and TOT methodologies and paired it with NTP staff, with the objective of developing the competencies of oblast-level officials who would run the e-TB Manager educational programs and also assure ownership of the intervention. While interactive methods of training are generally quite new to Ukraine’s public-sector health professionals, competency-based methodologies were employed. The TOT session was conducted over a 5-day period, with the first half focusing on adult learning methodologies and the second half on the core technical content of e-TB Manager. A series of six TOT sessions were organized and carried out, involving more than 100 key officials and the NTP program staff. Results of the trainings are summarized in Table 3 [37].

**Table 3:** Relevant Results (Ukraine)

By December 2015	Milestones
225,000 MDR-TB cases were in the e-TB Manager system	• TOT curriculum developed • E-TB Manager training scaled up nationally • Full transfer of the operation, administration, and development support of the e-TB manager was transferred from the program to the Government of Ukraine in 2015
More than 100 oblast officials reached over 1200 users in 26 oblasts	
Consistency between paper-based and electronically generated reports was about 99%	

In Mali, essential medicines and health commodities are frequently unavailable at various levels of the health system due to inefficient and poorly coordinated forecasting and quantification processes. To address these challenges, the program intervened to improve the individual capacity of senior leaders and administrators of key pharmaceutical institutions to redesign and roll out an improved Logistics Management Information System (LMIS). Training activities focused on strengthening the technical capacity of managers at all levels in the LMIS-related activities. During training activities, individuals and teams developed plans to support activity implementation. As a key follow-up activity, a joint government and program team visited several health districts to collect information on the status of individual implementation plans. Using coaching and supervision techniques, individual plans were evaluated by the team and shortcomings addressed. In many of the facilities, trained staff satisfactorily implemented at least one activity in their action plan, including: 1) good storage practices; 2) correct filing; and 3) management and submission of LMIS reports. Table 4 includes selected indicators related to training activities in Mali from 2013 to 2014.

**Table 4:** Relevant Results (Mali)

By December 2015	Milestones
1593 persons trained in pharmaceutical management	• Developed new LMIS that includes the community level • Developed training materials, tools, and job aids for the new LMIS • Increased percentage of stock records that correspond with physical counts for a set of indicator medicines in Ministry of Health storage and at health facilities, from 16% in 2013 to 42% in 2014 • Decreased percentage of warehouses with stock-outs of a pre-selected group of medicines for 3 days or more in the last 3 months, from 89% in 2013 to 66% in 2014 • Increased percentage of health facilities that completed and submitted an LMIS report for the most recent reporting period, from 7% in 2013 to 33% in 2014

### Proven approaches for individual capacity building

The program has applied participatory, team-oriented learning approaches that incorporate continuous feedback processes and group problem-solving by using local human resources and skillsets. These include task shifting structures, implementing continuous quality improvement measures, engaging the private sector, exploring online learning platforms, and placing an emphasis on effective knowledge sharing and exchange.

### Supportive supervision

The program assists governments and in-country counterparts to design and implement a supportive supervision plan and helps to conduct supportive supervision visits [38]. For example, in Lesotho, the program mentored 24 health care workers in the management of laboratory commodities, and conducted 135 supportive supervision visits to health facilities for LMIS and nutrition assessment counseling in three implementing districts, contributing to improved reporting rates, from 4 to 95% between two quarters [39]. In Swaziland 12 annual supportive supervision visits and mentorship in 131 health facilities contributed to increased LMIS reporting rate from 55% in 2012 to 95% in 2015. As a result of improved information quality, national authorities were able to make timely decisions and saved the government close to 6.25 million dollars in unnecessary procurement of medicines and commodities [40].

### Peer-to-peer learning mechanisms

Peer-to-peer learning mechanisms have been applied in specific circumstances. In Ukraine, the program facilitated a practical, interactive training approach rather than employing a traditional didactic method to train individuals in framework contracting in the pharmaceutical sector [41]. In Dnipropetrovsk oblast, authorities launched eight bids in framework contracting; six were successful. Thereafter the program brought the Dnipropetrovsk authorities to Poltava oblast, so that Poltava oblast authorities could hear lessons learned and ask relevant questions from Dnipropetrovsk authorities directly, rather than conducting a formal training program on framework contracting. By facilitating knowledge exchange among high-level government authorities, Poltava oblast authorities learned practical tips and successfully relaunched the tender process in framework contracting after a failed first attempt. The program facilitated a similar approach for high-level government officials in Bangladesh to learn from their peers in India for managing complex World Bank funded selection and procurement of medicines and commodities; and facilitated a partnership with the Korean International Cooperation Agency to enable peer learning with the Korean Ministry of Food and Drug Safety in medicines regulatory systems strengthening.

### Electronic information sharing

The program’s approach to capacity building recognizes the growing importance of electronic and new media for widespread access to learning and knowledge exchange [42]. New courses on good governance in the management of medicines and a two-part course on antimicrobial resistance are available on the global health eLearning center [43]. The World Health Organization’s dedicated information portal on Essential Medicines and Health Products has more than 5000 pharmaceutical management related documents [44]. In collaboration with the South Africa program, the University of Western Cape developed an online rational medicines use module [45]. An online course for the program’s electronic quantification and early warning system (Quan TB) provides access to learning resources with user guides available in six languages [46].

### Team-based learning approaches

To address critical leadership, management and governance skill gaps in South Africa [47], the program applied the Pharmaceutical Leadership Development Program (PLDP). The PLDP brings together health care professionals, including clinicians, pharmacists, facility managers, and operational managers, to strengthen their leadership, management, and governance skills, while engaging them in analyzing a persistent challenge they face at the health facility they serve. Adapted from Management Sciences for Health’s Leadership Development Program [48–50], the PLDP is designed to strengthen leadership, governance, and management capacity for health managers in public health service. The PLDP adaption includes additional content on legislation, ethics, governance, financial management, and human resources. It combines pharmaceutical management knowledge and sound leadership practices to better equip pharmacy managers to respond to challenges in their workplaces (Table 5).

**Table 5:** Selected Results from the Pharmaceutical Leadership Development Program (South Africa) [85]

Priority Areas	Province	Facility	Results
Waiting time	Western Cape	Kraaifontein Community Health Centre	Reduced average patient wait time at the pharmacy from 41 to 19 min over a 6 month period
Ensuring medicine accessibility	KwaZulu-Natal	Umzinto Primary Healthcare Clinic	Reduced the defaulter rate of patients collecting pre-dispensed chronic medicine from 28 to 23%
	Eastern Cape	Midlands Hospital and nearby clinics	Developed referral system which facilitated delivery of chronic diseases medicines from Midlands Hospital to feeder clinics
Improving medicine supply management	Eastern Cape	Cecilia Makiwane Hospital	Implemented a batch management system that cut the percentage of money wasted due to expired stock from 3.8% (as a percentage of expenditure) in April 2012 to 0.7% in June 2012, which is in keeping with international norms
	KwaZulu-Natal	Multiple clinics	Reduced the quantity of expired stock from 3.4% to less than 0.5% of stock holding
Ensuring compliance with standards	KwaZulu-Natal	Stanger, Montebello hospitals and Sundumbili CHC	Improved compliance with standard treatment guidelines for prescribing non-steroidal anti-inflammatory agents from 57 to 94%, 60 to 68%, and 37 to 67%, respectively
	North West	10 primary health care facilities	Increased compliance with national core standards from 33 to 77% by developing SOPs, distributing reference manuals, and building capacity in good pharmacy practice and medicine supply management
Ensuring rational use of medicines	North West	Joe Morolong Memorial Hospital	Average number of patients initiated on isoniazid preventive therapy increased from 3 to 8 per month
	North West	Four facilities in Bojanala District	Increased reporting of adverse drug events from 26 to 45%
	KwaZulu-Natal	Imbalenhle Community Health Centre	Reduced inappropriate prescriptions by 53%

An external, independent evaluation of the program stated “the people and institutions [in South Africa] who received this capacity building [including the PLDP] and tools [are] independently capable of managing and making improvements to their programs with minimal technical assistance […] from the program” [51]. With long-term sustainability in mind, the PLDP/LDP approach has been institutionalized at district and facility levels [52]. Several trained teams have continued to scale up their initial interventions. The management of team mentoring activities has been transitioned from the program to sub-district level teams.

### Survey findings from trainees in Bangladesh and Ethiopia

**Fig. 3:**
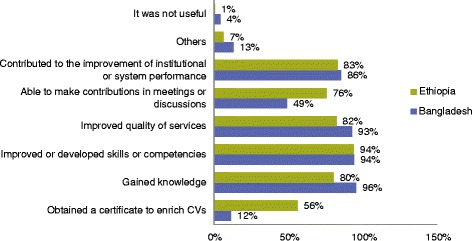
Feedback on training: results from Ethiopia (*N* = 153) and Bangladesh (*N* = 69)

Over 94% of respondents indicated that they have improved or developed skills or competencies as a result of the program’s training activities (Fig. 3).

In Bangladesh, an example of systems performance improvements include improved efficiency of supply chain systems in more than 29,000 service delivery points, improved use of data for decision making in 488 sub-districts, and greater customer satisfaction [53, 54]. One respondent stated the following in relation to LMIS [55] training efforts:

“[A] *higher authority is now aware, due to the online report, of increased transparency and quality of work; able to identify issues and make timely decisions; improving data quality in LMIS reports though improved inventory management (takes less time, get online* [and] *on to server easily); timely recovery of information from archive to satisfy information seekers,* i.e.*, audit; etc.*”

Key informant interviews with supervisors cited staff performance improvements on reporting data and report preparation, including reduced time required to complete a reporting task and improved accuracy in data entry and analysis. Another government official from Bangladesh’s Directorate General of Drug Administration (DGDA) overseeing medicines registration and pharmacovigilance activities remarked on the benefits of training approaches linked to improved quality of work or performance of the system:

“*Established a system for pharmacovigilance; publish regular medicine safety reports and newsletters; monthly field visits; uploading ADR-related data [adverse drug reactions] through Vigiflow system. Adverse Drug Reaction Monitoring cell of DGDA was awarded the 120th full membership of the WHO International Drug Monitoring Centre.”*

**Fig. 4:**
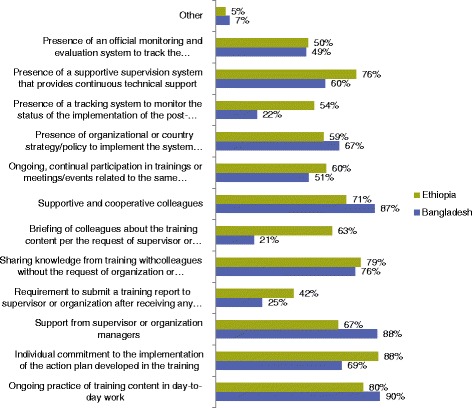
Post-training factors that contributed to the results of the training in Bangladesh (*N* = 69) and Ethiopia (*N* = 153)

Figure 4 indicates trainee reported post-training factors that contributed to the achievement of results. A small number of individuals indicated the reasons for training not being helpful (Table 6). Among respondents in both countries, the factors that promoted ongoing use of skills in daily on-the-job activities and those that incorporated group sharing and learning were mentioned the most frequently. Supportive supervision structures that enabled the implementation of post-training action plans were also frequently mentioned. One respondent in Ethiopia commented on the importance of having ongoing, interactive training to continue to move programmatic activities forward:

**Table 6:** Reasons why training was cited as not helpful

Reason provided by trainees surveyed	Bangladesh (*N* = 69)	Ethiopia (*N* = 153)
Training or capacity-building approach was not interesting	3	5
Training content was too challenging to understand and/or put into practice	2	9
Training content or technical area of the training did not correspond with the participants’ work	3	3
Appropriate environment was not in place to enable participants to use the knowledge gained during the training	2	5

“*One can mention APTS* [Auditable Pharmaceutical Transaction and Services] [56] *where, with the creative approaches of* [the program], *10 hospitals could be able to start APTS shortly after APTS training, unlike the classic trainings we know where staffs see training as means of a retreat.* [The program’s] *mentoring* […] *serves as a recipe for moving best practices forward* …”

**Fig. 5:**
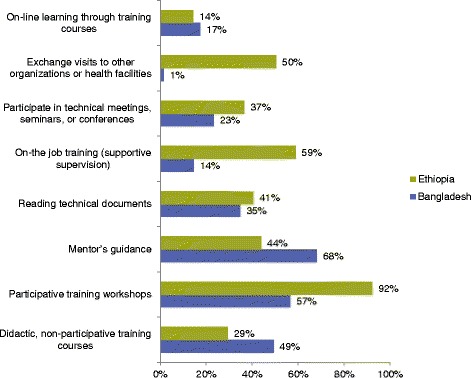
Top eight preferred learning methods, as chosen by survey participants in Bangladesh (*N* = 69) and Ethiopia (*N* = 153)

Overall, the majority of the respondents cited systems-related support mechanisms and strong interpersonal support as important components related to the success of training efforts. Figure 5 provides a summary of the top eight preferred learning methods (see Additional file 2 for the entire list). The detailed reports for both Bangladesh and Ethiopia provides further information on the training approaches used and insights from key informant interviews [57].

## Discussion

We provide a unique global implementer perspective regarding the variety of training approaches in the pharmaceutical system applied by country programs that fosters country ownership and sustainability consistent with other approaches [58]. Case-based learning, practice and feedback, and repetitive interventions such as post-training action plan, supportive supervision and mentoring approaches are effective, evidence-based training techniques applied by the program [59]. Individual capacity building efforts that include repetitive delivery and real-life case based trainings delivered positive results. South Africa’s experience with the PLDP demonstrates that ongoing work-place based training that encourages people to solve real life work-related challenges is an effective, case-based training modality. Twenty years of lessons on capacity building from the World Bank Institute’s Flagship Program also found that “*rather than a one-way dumping of information through repeated PowerPoint sessions,*” case-based learning and team approaches are crucial for learning and health systems improvement [60]. The PLDP in South Africa and implementation of post-training action plans in other countries applied similar principles to the monitoring-training-planning approach that emphasizes responsibility for implementing the pharmaceutical management practices learned in the hands of local staff [61]. Individual capacity training design tailored to the work environment and local context permit staff to receive ongoing and consistent feedback through multiple interventions with ongoing support from supervisors and peers [62].

Ensuring that strong systems are set up to bolster training activities is critical for those who are implementing individual capacity building efforts. Respondents from Bangladesh and Ethiopia perceived that both interpersonal support networks such as supportive supervision, and systems-level support mechanisms, such as aligned national and/or subnational strategies, improve the results of training as similar to the experience in Angola [63]. Yet, supportive supervision and mentoring which cost an annual average of US $ 1.2 million in just one district of South Africa for instance, must be adequately funded and institutionalized to mitigate the risk of not being planned and implemented [64]. Based on the experience from Mali, Namibia, South Africa and from other countries, the program ensured that supportive supervision structures and mentorship have been institutionalized with appropriate management structures. National authorities have been sensitized to secure funding from domestic resources or from the global fund grants for post-training follow-up initiatives.

Tailoring training activities to fit the local context is imperative for successful individual capacity building. Among the 12 selected countries reviewed, training was provided in several local languages: Amharic, Bangla, French, Portuguese and Ukrainian by local staff with the subject matter expertise frequently in collaboration with national and regional authorities. Over the years, the program took substantial efforts to have local staff deliver training and technical assistance interventions. The latter was acknowledged as “ethically sound” and culturally sensitive by leading educators at the Consortium of Universities for Global Health [65]. For specific topics that required international technical expertise such as pharmaceutical services, pharmacovigilance, regulatory systems strengthening, medicines registration, health technology assessment or digital health initiatives, interpretation would be provided in concert with rapid accessibility of translated training materials and job aids.

High turnover of staff challenges efforts to increase individual capacity of the workforce because training efforts alone are not sufficient to address larger issues relating to long-term recruitment and retention challenges. The Latin America model on facilitated distance learning for pharmaceutical services managers for continuous professional development may be replicated in the context of right enabling conditions and contribute to retention of health workers [66]. Interventions must involve local institutions in the design and delivery of content for both pre-service and in-service training as well as web-based methods where feasible [67]. The Ethiopia program collaborated with several university teaching hospitals as a strategy to ensure sustained local capacity of pharmacists and health workers for the APTS intervention and supportive supervision activities [68, 69].

Our program experience has implications for those considering the future of pharmaceutical workforce development and training, given the High-Level Commission on Health Employment and Economic Growth’s 10-point recommendations on investing in the health workforce to ensure progress towards the Sustainable Development Goals and to achieve Universal Health Coverage [70]. Effective pre-service training potentially reduces the need for future large-scale and expensive in-service trainings [71]. The program’s experience demonstrate that competency based pre-service training will prepare students with a critical foundation of knowledge and skills required in the pharmaceutical system [72–74]. Sometimes, new topics such as pharmacovigilance [75], electronic medicines registration processes [76, 77], clinical pharmacy services [78, 79], patient-centered pharmaceutical care [80, 81], improving antibiotic prophylaxis [82] or medicines benefits package for universal health coverage [83] require comprehensive in-service and on-the-job training. The program’s external evaluation found that national pharmaceutical system stakeholders are “*increasingly concerned about the development of sustainable human capacity to support the growing demand for more and more sophisticated pharmaceutical services*” and acknowledged the program’s multi-pronged efforts [51]. The program’s efforts complement the Nanjing statement on needs based approach through 13 global pharmaceutical workforce development goals for improving global health [84].

### Limitations

Our selected review of the program’s 12 implementing countries may be subject to selection bias because the program operated in more than 24 countries. Differences in training approaches and resulting data variation across countries and time contributes to limitations of this review. As a result, we are unable to compare data across countries. Moreover, because training events were developed for country-specific needs, training-related indicators were not applicable across all countries. Therefore, there was no way to compare training activities across countries and to generate trends at a global level. We do not elaborate the rationale in a country program’s choice of a certain individual capacity building approach nor describe what did not work because there were multiple country-specific contextual factors that were not within the scope of this selected multi-country review. Because our focus was a selected review on modalities of training approaches in multiple countries based on the first 3 years of program implementation, we do not provide results on patient or health related outcomes associated with specific training interventions. The Bangladesh and Ethiopia trainee surveys included small sample sizes due to time and funding constraints. Yet our paper offers a unique implementer perspective supported with references to various country project documents.

## Conclusion

Based on Potter and Brough’s model, the program utilized a hierarchy of individual capacity building approaches that address the needs for tools (contextualized training curricula, job aids), skills (competency- and needs- based training design), and staff and facilities (training and post-training support). These three tiers build the foundation of improving the pharmaceutical system capacity. Program implementers must systematically collaborate with local institutions to provide coordinated training efforts that fill current skill shortage gaps, meet local needs in the pharmaceutical system, and promote the ownership of human capacity development interventions. Robust monitoring and evaluation efforts that are adequately funded must accompany individual capacity building programs to ensure that effective training methods are scaled up while those that are weak or ineffective are discontinued. Finally, training alone is not sufficient to address major human resource shortages and skill gaps. It must be paired with other system-level efforts to improve recruitment and retention of professionals.

## Additional files

Additional file 1: Detailed methodology for surveys and key information interviews in Bangladesh and Ethiopia. (DOCX 30 kb)

Additional file 2: Learning Methods that Worked Best for the Respondents According to their Experiences. (DOCX 18 kb)

## Abbreviations

ACPE: Accreditation Council for Pharmaceutical Education
APTS: Auditable Pharmaceutical Transaction and Services
B Pharm: Bachelor of Pharmacy
DGDA: Directorate General of Drug Administration [Bangladesh]
DR Congo: Democratic Republic of the Congo
LMIS: Logistics Management Information System
NTP: National Tuberculosis Program
PLDP: Pharmaceutical Leadership Development Program
TOT: Training of trainers
USAID: United States Agency for International Development
WHO: World Health Organization
