# Supplementary material for: Individual capacity-building approaches in a global pharmaceutical systems strengthening program: a selected review
Source: J Pharm Policy Pract. 2017 May 8;10:16. doi: 10.1186/s40545-017-0104-z (PMC5422928; doi:10.1186/s40545-017-0104-z)
Supplement: Supplementary file 1 — Detailed methodology for surveys and key information interviews in Bangladesh and Ethiopia. (DOCX 30 kb) [file 40545_2017_104_MOESM1_ESM.docx]

Additional file 1: Detailed Methodology for Surveys and Key Information Interviews In Bangladesh and Ethiopia

**Ethiopia**

***Methods***

This review applied a cross-sectional study approach. Four types of respondents were included in the review: trainees and trainers; government officials and trainees’ supervisors; physicians that work with pharmacists for clinical pharmacy services; and Ethiopia program staff. The trainees’ responses were collected through a self-administered, semi-structured questionnaire. The trainees were randomly selected from the base total of 5,037 people who were trained between December 2011 and September 2015 in the technical areas listed in table B1. The government officials and supervisors were selected based on their oversight responsibilities at the central level and the trainees’ facilities. They were interviewed using an open-ended questionnaire. The physicians who worked with pharmacists for CP services were selected from six hospitals; they responded to a self-administered, semi-structured questionnaire. The total numbers of respondents and response rates were 153 trainees out of 222 contacted (69%), including 149 trainees, and 4 trained as trainers (table B1); 7 physicians (100%) that work with pharmacists for CP services; and 19 government officials or trainees’ supervisors out of 33 who were interviewed (58%). The 153 trainees were trained between 2012 and 2015 (table B2); they came from 5 types of organizations located in 8 regions. The majority of them came from health facilities. Nine program Ethiopia team members self-responded to an open-ended questionnaire. The interviews and questionnaire administration were completed during November and December 2015.

Table B1. Types of Training, Sample Sizes, and Response Rates

|  | **Types of training** | **Sample size** | **Number of trainees who responded** | **Number of trainers who responded** |
| --- | --- | --- | --- | --- |
|  | Waste disposal familiarization | 11 | 7 |  |
|  | Ethics | 4 | 4 |  |
|  | Leadership & management | 4 | 3 |  |
|  | Antimalarial drug management | 12 | 0 |  |
|  | Antimalarial resistance | 10 | 6 |  |
|  | Antiretroviral therapy (ART) (in-service) | 14 | 14 | 2 |
|  | ART (pre-service) | 9 | 5 |  |
|  | Clinical pharmacy | 11 | 9 |  |
|  | Drug information services | 7 | 5 |  |
|  | Rational drug use related | 18 | 16 |  |
|  | Ethiopian Hospital Reform Implementation Guideline (HRIG) | 3 | 1 |  |
|  | Midlevel staff | 4 | 4 |  |
|  | Pharmacovigilance (PV) | 2 | 0 |  |
|  | Electronic dispensing tool (EDT) | 6 | 4 |  |
|  | Standard operating procedure (SOP) manual | 41 | 23 |  |
|  | Auditable Pharmaceutical Transactions and Services (APTS) | 66 | 48 | 2 |
| **Total** | | **222** | **149** | **4** |
| **Response rates** | |  | **69%** | |

Table B2. Respondents’ Type of Organization

| **Type of organization** | **Numbers of respondents** |
| --- | --- |
| Government entity | 26 |
| Health facility (HF) | 114 |
| Media | 3 |
| Private (suppliers & HF) | 8 |
| Academic | 2 |
| **Total** | **153** |

Table B3. Years in Which Respondents (Trainees and Trainers) were Trained

| **Year of the training** | **2011** | **2012** | **2013** | **2014** | **2015** | **N/A** | **Total** |
| --- | --- | --- | --- | --- | --- | --- | --- |
| Number of respondents | 5 | 40 | 28 | 13 | 36 | 31 | 153 |

***Limitations***

Due to time and financial constraints, the assessment was conducted via self-administered questionnaires given to the trainees and trainers, physicians, and program staff. The government officials and HF managers were interviewed by local staff in Ethiopia. Therefore, it was impossible to conduct in-depth interviews with the majority of the respondents to gain more insight. The review aimed to gain an understanding of training methods and the results of training for individual capacity building. It was not feasible to do causal analysis of the contribution of training alone to the broader system performance because there were a series of interventions undertaken by the program, of which training was only one part. Therefore, other factors that helped the trainee respondents achieve their results are discussed.

**Bangladesh**

***Methods***

This review was conducted through a desk review of several training reports and the program’s Program Year (PY) 4 Annual Report. Key informant interviews were also conducted with Bangladesh program staff, a limited number of trainees, and government officials and health-facility managers. The local program staff in Bangladesh was interviewed in four portfolio-basis groups during conference calls conducted in November 2015 by a program headquarters staff member based in the US. An open-ended questionnaire was used. A local consultant was hired to conduct interviews with trainees using a semi-structured questionnaire, and with the government officials and health-facility managers using an open-ended questionnaire. These interviews were conducted in January 2016. Sixty-nine trainees from the public and private sectors were conveniently selected from 29 upazilas (see “Limitations” section) according to the technical areas of training (tables B4 and B5) that had been conducted between January 2012 and September 2015 (table B6). Five upazila health officials and health-facility managers who oversaw supply management, procurement, TB project and pharmaceutical regulatory affairs were selected from five upazilas, as well as six central-level officials in Dhaka, where the central government is based.

Table B4. Types of Training and Sample Sizes

| **Type of training** | **DGDA** | **Logistics** | **Procurement** | **TB** |
| --- | --- | --- | --- | --- |
| Adverse Drug Reaction Monitoring Cell | 2 |  |  |  |
| Focal points for PV | 2 |  |  |  |
| PV Workshop | 2 |  |  |  |
| Medicine Registration | 3 |  |  |  |
| Good Manufacturing Practices Inspection | 3 |  |  |  |
| Logistics Management Training (LMT) |  | 12 |  |  |
| Upazila Inventory Management System (UIMS) Training |  | 12 |  |  |
| Master Trainer (for LMT) |  | 2 |  |  |
| Bidders Orientation |  |  | 5 |  |
| Framework Agreement |  |  | 3 |  |
| Procurement Post-Review & Audit Trial |  |  | 2 |  |
| Basic training on procurement of Goods and Services (conducted by SETYM International^[[1]](#footnote-1)^) |  |  | 2 |  |
| eTB Manager |  |  |  | 12 |
| multidrug-resistant TB (MDR-TB) |  |  |  | 2 |
| TB Logistics Management Information System (LMIS) |  |  |  | 3 |
| Master Trainer (for TB) |  |  |  | 2 |
| Total | 12 | 26 | 12 | 19 |

Table B5. Respondents’ Type of Organization

| **Type of organization** | **Numbers of respondents** |
| --- | --- |
| Government entity | 43 |
| Health facility (HF) | 20 |
| Private (suppliers & HF) | 6 |
| **Total** | **69** |

Table B6. Year in Which Respondents (Trainees and Trainers) Were Trained

| **Year of training** | **2012** | **2013** | **2014** | **2015** | **Total** |
| --- | --- | --- | --- | --- | --- |
| Number of respondents | 2 | 14 | 36 | 17 | 69 |

***Limitations***

Due to time and budget constraints, the sample size for the trainees was significantly reduced from over 200 to 69 who were traceable and easily accessible geographically. The review aimed to gain an understanding of training methods and the results of training for individual capacity building. It is not possible to do causal analysis of the broader system performance because the training was one of a series of interventions undertaken by the program. Therefore, other factors that helped the respondents achieve their results are presented and discussed.

1. SETYM International. <http://www.setym.com/en-ca/Home/Default.aspx>. [↑](#footnote-ref-1)
