# Supplementary material for: Individual capacity-building approaches in a global pharmaceutical systems strengthening program: a selected review
Source: J Pharm Policy Pract. 2017 May 8;10:16. doi: 10.1186/s40545-017-0104-z (PMC5422928; doi:10.1186/s40545-017-0104-z)
Supplement: Supplementary file 2 — Learning Methods that Worked Best for the Respondents According to their Experiences. (DOCX 18 kb) [file 40545_2017_104_MOESM2_ESM.docx]

# **Additional File 2: Learning Methods that Worked Best for the Respondents According to their Experiences**

|  | Bangladesh | | Ethiopia | |
| --- | --- | --- | --- | --- |
|  | N | % | N | % |
| Didactic, non-participatory training courses | 34 | 49% | 45 | 29% |
| Participatory training workshops | 39 | 57% | 141 | 92% |
| Mentor’s guidance | 47 | 68% | 67 | 44% |
| Reading technical documents | 24 | 35% | 62 | 41% |
| Participate in accredited continuous education program | 2 | 3% | 40 | 26% |
| On-the job training (supportive supervision) | 10 | 14% | 90 | 59% |
| Participated in supportive supervision | 1 | 1% | missing | missing |
| Have technical discussions with colleagues or other professionals | 4 | 6% | 50 | 33% |
| Participate in technical meetings, seminars, or conferences | 16 | 23% | 56 | 37% |
| Making presentations in a meeting or conference | 9 | 13% | 17 | 11% |
| Having the responsibility to train or mentor others | 1 | 1% | 31 | 20% |
| Learn from observing how my supervisor or colleagues do the work | 3 | 4% | 21 | 14% |
| Have job aids posted in my work station | 1 | 1% | 18 | 12% |
| Follow the instructions of guidelines or SOPs | 0 | 0% | 35 | 23% |
| Exchange visits to other organizations or health facilities | 1 | 1% | 77 | 50% |
| On-line learning through training courses | 12 | 17% | 22 | 14% |
| On-line learning through social media | 5 | 7% | 9 | 6% |
| Others | 2 | 3% | 1 | 1% |
| Total | 69 |  | 153 |  |
